# Nanostructural insights into Mongolian medicine Harigabri and its therapeutic efficacy for gastrointestinal diseases^☆^

**DOI:** 10.1016/j.fochx.2025.102838

**Published:** 2025-07-24

**Authors:** Galama Ang, Qiaoli Ren, Jun Ai, Gerile Aodeng

**Affiliations:** a College of Chemistry and Enviromental Science, Inner Mongolia Normal University, 81Zhaowudalu, Hohhot 010022, China; b Beijing National Laboratory for Molecular Sciences, Key Laboratory of Analytical Chemistry for Living Biosystems, Institute of Chemistry Chinese Academy of Sciences, Beijing, China.

**Keywords:** Mongolian medicine, Harigabri, Graphene oxide, Gastric ulcer

## Abstract

The structural and functional properties of Harigabri, a traditional Mongolian remedy for gastrointestinal disorders, were investigated using a range of advanced analytical techniques. Raman spectroscopy and X-ray diffraction provided complementary insights into the structures of both raw Harigabri and its ultrasonic extract. Fourier-transform infrared spectroscopy further characterized their chemical functionalities. Additionally, transmission electron microscopy revealed nanostructural features. Collectively, these methods demonstrated that Harigabri and graphene oxide (GO) share similar layered architectures. Based on these structural similarities, the study evaluated the antibacterial activity against gastrointestinal pathogens and the therapeutic efficacy against gastric cancer of both Harigabri and GO. This integrated approach offers a detailed understanding of Harigabri's unique nanostructure and highlights its potential, alongside GO, as a novel treatment modality for gastrointestinal diseases and gastric cancer.

## Highlights

• Advanced techniques like Raman, X – ray diffraction, FT - IR, and TEM used on Harigabri.
• Complementary insights on raw and extract's structure.
• Chemical functionality of Harigabri characterized by FT - IR.
• Nanostructural features of Harigabri revealed by TEM.
• Harigabri and graphene oxide have analogous layered architectures.
• Similarities between them are basis for further evaluation.

## 1 Introduction

Mongolian medicine, with its millennia-old heritage, demonstrated clinical efficacy, and abundant natural resources, holds tremendous potential for modern development. To improve the overall technical level and core competitiveness of Mongolian medicine, three critical challenges must be overcome: the identification and characterization of its active constituents, the establishment of comprehensive and standardized quality-control criteria, and the modernization of its dosage-form technologies (Bai, 2025; Song et al., 1999; Zhu, 2021). Harigabri (also known as Karina GA), first recorded in 《Bai Jingjian》 (Song, 2004), is a traditional Mongolian medicinal charcoal obtained by fully carbonizing the dried feces of *Sus scrofa* Linnaeus. The preparation of Harigabri involves several sequential steps: harvesting, removing impurities, drying, high-temperature calcination, cooling, collection, crushing, and standby (Chen et al., 2009; Du et al., 2018). Bitter in taste and pungent and warm in nature, it is traditionally believed to calm “Xieri,” promote digestion, eliminate “Stickiness,” and dispel ruffians. It is primarily used for conditions such as dyspepsia and other disorders attributed to “Xieri” and “Stickiness.” Black ice tablet is a distinctive medicinal material in Mongolian medicine, with a clinical history spanning thousands of years. It is noted for its remarkable efficacy and relatively few adverse reactions (Luo, 2006). However, owing to its unique origin, complex components, and as-yet unclear mechanism of action, comprehensive studies on its structural features, physicochemical properties, and pharmacodynamic mechanisms remain scarce.

In this study, we performed comprehensive structural characterization of Harigabri and found that it exhibits features characteristic of graphene derivatives. We then used ultrasonic extraction to isolate its principal components, which, upon analysis, retained the graphene-like layered architecture and revealed an enhanced nanoscale sheet morphology. Building on these insights, we focused our subsequent investigations on graphene oxide (GO), a prototypical graphene derivative, to elucidate its pharmacodynamic mechanisms—specifically, its antibacterial activity against gastrointestinal pathogens and its therapeutic efficacy in a gastric ulcer model. While previous research has established Harigabri's broad-spectrum antibacterial effects, the specific active constituents and their structure–activity relationships have not been clearly defined. This work was designed to address this knowledge gap by combining structural analyses with functional evaluations.

## 2 Materials and methods

### 2.1 Processing method

**Fig.1:**
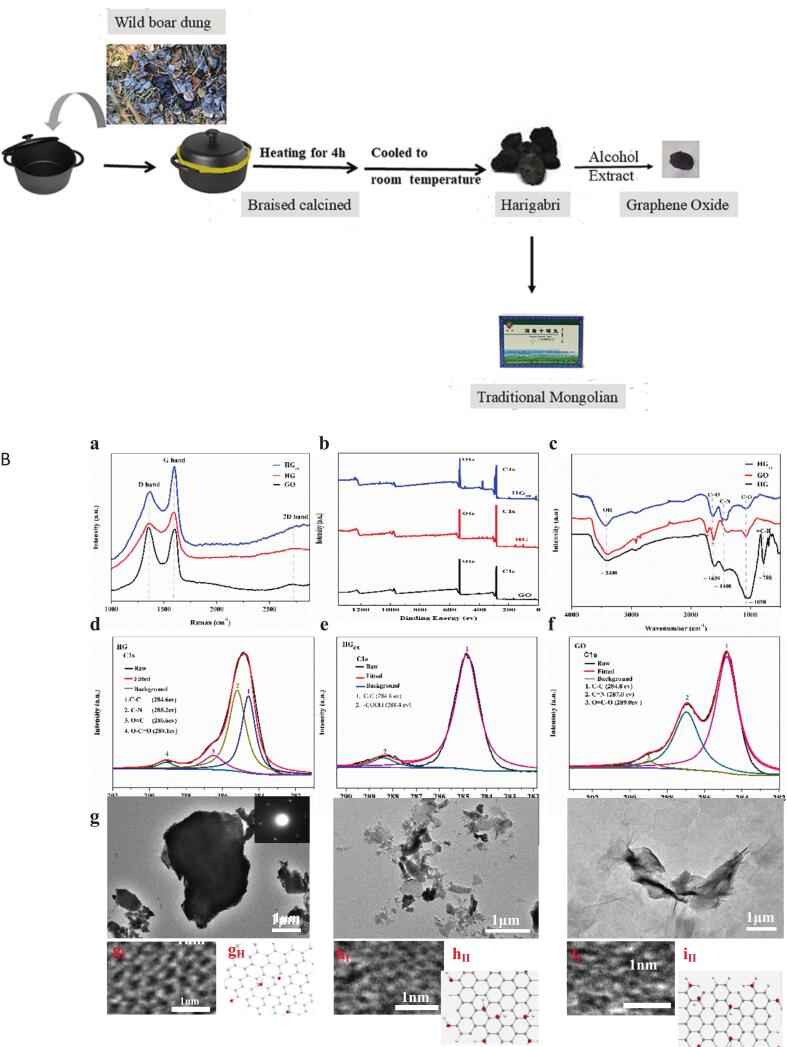
A. Overview of Harigabri processing and Ethanol extraction; B. Structural characterization of Harigabri (HG), its ultrasonic extract (HGex), and graphene oxide (GO). (a) Raman spectra of HG, HG_ex_, GO. (b) XPS Energy spectra of HG, HG_ex_, GO. (c) FT-IR spectra of HG, HG_ex_, GO. (d) XPS energy spectra of HG C1s. (e) XPS energy spectra of HG_ex_ C1s. (f) XPS energy spectra of GO C1s. (g) HG TEM data (g_I,_ g_II_) and molecular model. (h) HG_ex_ TEM data. (h_I,_ h_II_) and molecular model. (i) GO TEM data. (i_I,_ i_II_) and molecular model.

Mongolian medicine Harigabri is supplied as a finished product by Inner Mongolia International Mongolian Medical Hospital and can be directly used as medicine. It is prepared by high-temperature calcination of boar manure. The specific process is shown in Fig. 1. We extracted the active ingredient GO from Harigabri (Song et al., 1999; Zhu, 2021).

The ultrasonic extraction process of Mongolian Medicine (HGex) is as follows: First, 20.0 g of finely ground Harigabri was mixed with 200.0 mL of 90 % ethanol and subjected to ultrasonic treatment for 12 h. After standing for several hours, the supernatant was removed. The precipitate was labeled as S1 and centrifuged; the supernatant after centrifugation was collected and labeled as L1.

Next, the centrifuged precipitate was recovered and mixed with S1. Then, 160.0 mL o f 90 % ethanol was added to the mixture. After ultrasonic treatment for 1 h, the mixture was allowed to stand for a few hours. During the standing period, large blocks of Harigabri that have not undergone ultrasonic treatment may cause unsonicated particles to aggregate, leading to the precipitation of certain substances in the mixture. The supernatant was then removed. The remaining precipitate was labeled as S2. Suspended particles were separated by centrifugation, and the supernatant was collected and labeled as L2.

The recovered precipitate was combined with S2 and suspended in 80.0 mL of 90 % ethanol. After ultrasound treatment for 1 h, the mixture was allowed to stand for several hours to promote sedimentation of any remaining Harigabri aggregates. The supernatant was removed and the precipitate was labeled S3. Suspended particles were separated by centrifugation; the supernatant was collected and labeled L3. The centrifuged precipitate was recovered and added to S3.

The supernatants L1, L2, a nd L3 were mixed and placed in a round-bottom flask. The mixture was concentrated on a rotary evaporator at a rotation speed of 70 rpm and heated at 50 °C. When approximately 5 mL of liquid remained at the bottom of the flask, rotary evaporation was stopped. The concentrated liquid was transferred to a clean beaker and dried at 60 °C for 12 h until completely dry. Finally, the dried extract was collected and stored for further analyses by Raman, X-ray powder diffraction (XRD), X-ray photoelectron spectroscopy (XPS), and transmission electron microscopy (TEM).

Synthesis of GO: One gram of graphite was accurately weighed and placed into a 250 mL flask, to which 23 mL of concentrated H₂SO₄ was added. Then, 3.0 g of KMnO₄ was slowly added under stirring at 200 rpm in an ice bath, maintaining the reaction temperature below 20 °C. The reaction was carried out in an oil bath at 40 °C for 0.5 h with stirring at 300 rpm. Next, 50 mL of water was added, the temperature was raised to 90 °C, and the mixture was reacted for 15 min. Subsequently, 150 mL of water and 5 mL of 30 % H₂O₂ were added dropwise, turning the reaction color from brown to yellow. The mixture was centrifuged to collect the precipitate, which was washed three times with 1:9 HCl (50 mL each time) to remove unreacted metal ions and impurities. The cleaned precipitate was air-dried, then resuspended in 300 mL of water to form GO dispersions. These dispersions were purified by dialysis for 1 week to remove residual acids and metal contaminants. The dialyzed GO was stirred overnight and ultrasonicated for 30 min. Finally, graphite particles were removed by centrifugation at 3000 rpm for 40 min.

### 2.2 General biological characteristics experiment

After weighing and labeling, the mice were randomly divided into nine groups: blank control group, aspirin model group (22.4 mg/20 g), positive drug omeprazole group (0.104 mg/20 g), low-dose traditional Chinese medicine group (0.78 mg/20 g), high-dose traditional Chinese medicine group (1.56 mg/20 g), low-dose traditional Chinese medicine group (7.8 mg/20 g), high-dose traditional Chinese medicine group (31.2 mg/20 g), low-dose graphene group (0.0078 mg/20 g), and high-dose graphene group (0.0312 mg/20 g), with 15 mice in each group.

Except for the blank group, all other groups were induced with aspirin by gavage for 14 days. On the 8th day, corresponding concentrations of the test substances were administered alongside continuous modeling. The blank and model groups received physiological saline by gavage, while the remaining groups were administered the respective test substances by gavage for 7 days. Animals were deprived of food and water prior to each aspirin gavage; specifically, water was withheld for 24 h before the final administration. Biological samples were collected 2 h post-dose for subsequent measurement of designated parameters.

Throughout the experiment, food intake and weight of the mice were observed daily. Compared with the model group, the treatment groups showed increased food intake and weight gain.

#### 2.2.1 Orbital blood collection in mice

The venous plexus at the corner of the eye was punctured using blood collection vessels, and blood was collected into 1.5 mL centrifuge tubes. The serum was separated, labeled, and frozen at −80 °C for later use.

#### 2.2.2 Measurement of gastric ulcer index and ulcer inhibition rate

The stomach was rinsed with normal saline and dried with filter paper. Ulcers were examined under a microscope. The ulcer index was calculated according to the modified Guth standard: punctate erosions (< 1 mm diameter) were assigned 1 point for every three lesions; linear bleeding ulcers (> 1 mm diameter) were scored by length and width—1 point per millimeter of length when <1 mm wide, 2 points per millimeter of length when ≥1 mm but <2 mm wide, and 3 points per millimeter of length when ≥2 mm wide.

Ulcer inhibition rate (%) = (ulcer index of model group – ulcer index of administration group) / ulcer index of model group × 100 %.

Ulcer area was calculated by measuring the maximum length and perpendicular diameter of the ulcer using a vernier caliper. The formula used was: area = length × width.

### 2.3 Material characterizations

Morphological and structural information of all samples was obtained using a Hitachi S-4800 scanning electron microscope and JEOL-2100F transmission electron microscope. Energy dispersive X-ray spectroscopy was employed to detect elemental composition and distribution on catalysts. XRD patterns were acquired using Cu Kα radiation (λ = 0.154056 nm) on a PANalytical X'Pert PRO instrument. XPS measurements were performed on an ESCALAB 250XL spectrometer. Nuclear magnetic resonance analysis was conducted using an Avance III HD 500 instrument from Bruker.

## 3 Results and discussion

### 3.1 Structural characterization of Harigabri, its extract and graphene oxide

To better understand the composition of Mongolian medicine Harigabri, its extract, and GO, their basic structures were characterized by Raman spectroscopy, XPS, FTIR, and TEM. The results are shown in Fig. 1. Fig. 1A shows the D, G, and 2D graphene peaks near ∼1350, ∼1580, and ∼ 2700 cm^−1^, respectively, for Harigabri, Harigabri extract, and GO. The G peak originates from the sp^2^ stretching vibration of carbon, associated with the doubly degenerate phonon mode at the center of the Brillouin zone with E₂g symmetry. The D and 2D peaks arise from the second-order double resonance Raman scattering process in the first Brillouin zone; the 2D peak originates from the in-plane transverse optical phonon branch at the zone boundary, whereas the D peak originates from the phonon branch near the K point and requires defects to be activated. Generally, the D, G, and 2D peaks reflect the defect structure, degree of order, and number of layers in graphene. These data show that Mongolian medicine Harigabri and its extracts exhibit a GO structure with prominent oxidized defect features.

Furthermore, the full XPS spectrum in Fig. 1B and corresponding high-resolution spectra in Figs. 1C–F of the extract and GO reveal abundant oxygen elements and oxygen-containing functional groups. These include C—H, C—N (285.2 eV), C=O (286.6 eV), and O–C–O (289.1 eV) in HG; –COOH (288.4 eV) in HGex; and C=N (287.0 eV) and O=C–O (289.0 eV) in GO. The presence of these functional groups enhances biocompatibility and bactericidal properties (Xie et al., 2020).

TEM images in Figs. 1G–I show that the morphology of Mongolian medicine Harigabri and its extract resembles that of GO. Selected area electron diffraction patterns acquired via TEM display well-defined hexagonal rings indicative of an ideal, defect-free lattice. High-resolution TEM further confirms that both Harigabri and its ultrasonic extract possess a six-membered-ring layered architecture analogous to GO. Although the lattice fringes of Harigabri extract and GO closely coincide, the extract exhibits a higher density of lattice defects than Harigabri, likely due to ultrasonic cavitation and mechanical action during extraction. The defective oxygen-containing functional groups contribute to enhanced antibacterial effects (Lu et al., 2019).

Harigabri, a traditional Mongolian remedy for gastrointestinal disorders, has a long history of clinical use despite limited scientific characterization of its chemical constituents, nanostructural features, and pharmacodynamic mechanisms. In this work, both Harigabri and its ultrasonic extract exhibit the characteristic layered architecture and oxygen-functional groups typical of GO. GO is known for its excellent antibacterial effects due to its small size and unique surface structure. Consequently, various GO-based antimicrobial products—including water vessels, textiles, and wound dressings—have been commercialized. However, its unique size, morphology, and surface structure also raise significant biosafety concerns, confining GO's clinical applications to the experimental stage.

**Fig. 2:**
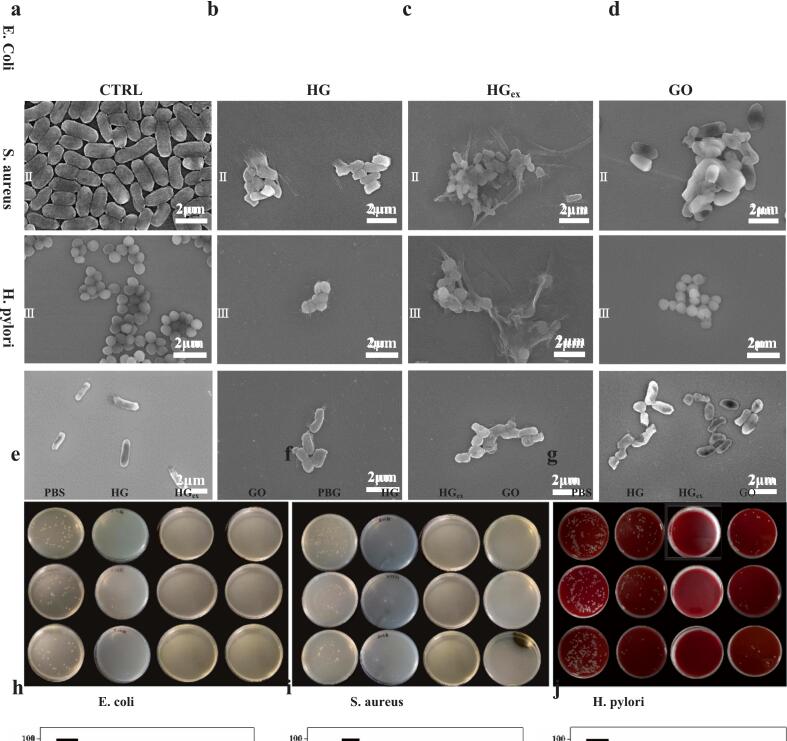
Antibacterial effects of Harigabri (HG), its ultrasonic extract (HGex), and graphene oxide (GO) against gastrointestinal pathogens. (a) SEM micrographs of *E. coli*, *S. aureus*, and *H. pylori*. After incubation with PBS (blank control). (b) SEM images of the three bacterial species following treatment with HG (b), HGex (c), and GO (d), respectively, showing progressive membrane damage. (e–g) Representative agar-plate photographs of *E. coli* (e), *S. aureus* (f), and *H. pylori* (g) after treatment with PBS, HG, HGex, or GO. (h–j) Quantitative survival rates of *E. coli* (h), *S. aureus* (i), and *H. pylori* (j) following each treatment vs PBS control.

Based on these experimental results and the traditional use of Harigabri for gastrointestinal diseases, we hypothesized that its mechanism of action may involve interactions with gastrointestinal bacteria. Therefore, in this study, we selected *Escherichia coli* (*E. coli*) and *Staphylococcus aureus* (*S. aureus*) as representatives of intestinal bacteria, and *Helicobacter pylori* (HP) as a representative of gastric bacteria to evaluate the antibacterial properties of Mongolian medicine Harigabri, its extract, and GO. The experimental results are shown in Fig. 2. In Fig. 2A, the control groups of *E. coli*, *S. aureus*, and HP maintained intact cell structures. Upon incubation with Harigabri, its extract, and GO, the cell membranes of all three bacteria were disrupted, and cell integrity was severely compromised, as shown in Figs. 2B–D. Plate colony growth and corresponding survival rate analyses further demonstrated that Harigabri, its extract, and GO exert potent antibacterial effects against *E. coli*, *S. aureus*, and HP (Figs. 2E–G). These findings confirm the presence of GO–like features in HGex and demonstrate that Harigabri and its extract exhibit antibacterial effects comparable to GO against key gastrointestinal pathogens. Collectively, Mongolian medicine Harigabri, its extract, and GO significantly reduced the growth rates of *E. coli* and *S. aureus* compared to the control sample (Majidi et al., 2019).

### 3.2 Antibacterial mechanism of Harigabri, its extract, and GO

GO exerts its antibacterial activity primarily by inducing oxidative stress, which disrupts cellular redox homeostasis, impairs metabolic processes, and ultimately inactivates bacterial cells. Oxidative stress occurs mainly via reactive oxygen species (ROS)-dependent and ROS-independent pathways. The ROS-dependent pathway involves the excessive accumulation of ROS in cells, such as hydrogen peroxide (H₂O₂), superoxide anion (O₂^−^), hydroxyl radical (OH·), or singlet oxygen (^1^O₂). GO can also oxidize various reducing enzymes and biomolecules, such as ascorbic acid, which is an effective antioxidant crucial for cell cycle redox reactions.

**Fig. 3:**
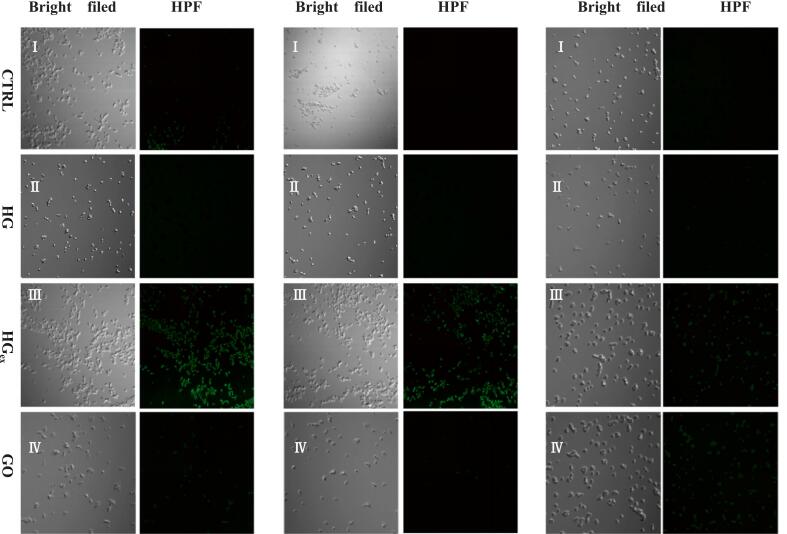
Fluorescence micrographs of ROS in *E. coli*, *S. aureus* and *H. pylori* after interaction with PBS, HG, HGex, and GO. I, II, III, and IV represent corresponding bright field micrographs.

In this study, we used dichlorodihydrofluorescein diacetate (DCFH-DA) as a fluorescence probe to detect intracellular ROS levels in bacteria. DCFH-DA is non-fluorescent and freely penetrates the cell membrane, where it is hydrolyzed by intracellular esterases to 2′,7′-dichlorodihydrofluorescein (DCFH), which cannot cross the membrane. If ROS is present within the cell, DCFH is oxidized to 2′,7′-dichlorofluorescein, which emits green fluorescence. The fluorescence intensity correlates positively with intracellular ROS levels. Fig. 3 shows that bacteria treated with Harigabri, Harigabri extract, and GO all exhibited green fluorescence, with the Harigabri extract group displaying the strongest signal. These results indicate that Harigabri, its extract, and GO promote ROS production in bacteria, thereby inactivating them (Yao et al., 2018). Collectively, the experimental results support that oxidative stress is a principal mechanism underlying the antimicrobial activity of Harigabri, HGex, and GO (Ojha et al., 2025).

### 3.3 Therapeutic effect of Harigabri, its extract, and GO on gastric ulcer in mice

**Fig.4:**
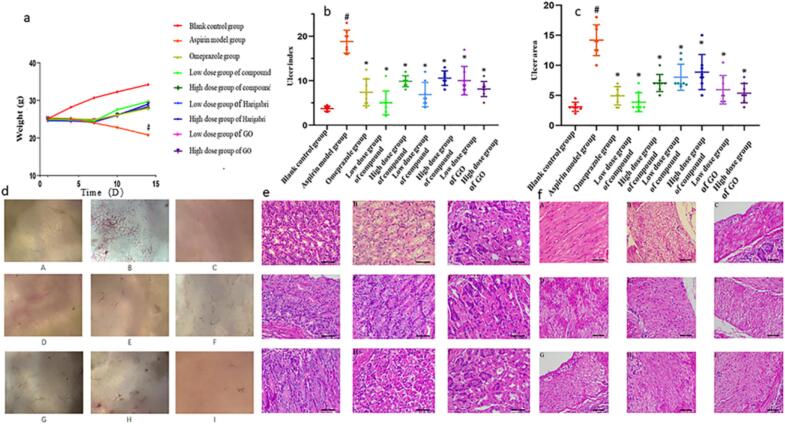
(a) Effects of each test substance on body weight changes in gastric ulcer mice (# significantly different from the blank group, *P* < 0.05). (b) Effects of each test substance on ulcer index in gastric ulcer mice (# significantly different from the blank group, P < 0.05, * significantly different from the model group, P < 0.05) (c) The effect of each test substance on the ulcer area of gastric ulcer mice (# significantly different from the blank group P < 0.05, * significantly different from the model group P < 0.05) (d) Pathology of gastric villus tissue in each group of mice (cytoplasmic vacuolization; cavity) (e) Pathology of gastric villous tissue in each group of mice (I, II, III, IV, V, VI represent blank group, aspirin modeling group, omeprazole positive drug group, HG administration group, HGex administration group, GO administration group, respectively) (f) The normal control group had uniform staining of the muscle layer, tightly arranged cells, intact cytoplasm, and clear and regular patterns.

Mongolian medicine Harigabri not only inhibits *E. coli*, *S. aureus*, HP, and other gastrointestinal bacteria but also demonstrates a significant therapeutic effect on aspirin-induced gastric ulcers in mice, as shown in Fig. 4. Fig. 4a presents the therapeutic effects of Harigabri (HG), its ultrasonic extract (HGex), and GO in the aspirin-induced gastric ulcer model. The results showed that the body weight of mice in the model group significantly decreased over 14 days compared with the blank group. In contrast, the weight in the other treatment groups decreased slightly within 7 days but gradually increased after administration, with no significant difference compared to the blank group.

The stomachs of the mice were then examined under a stereomicroscope to assess the degree of gastric ulceration in each group, as shown in Fig. 4b and c. Quantitative analyses indicated that the ulcer index of the model group was significantly higher than that of the blank group, consistent with experimental expectations. Statistical analysis showed that compared to the model group, the ulcer index and ulcer area in the treatment groups decreased significantly, with reductions close to those observed in the positive drug and blank groups (*P* < 0.05), indicating that each treatment exerted a strong therapeutic effect on aspirin-induced gastric ulcers.

At the end of the experiment, mice were euthanized via cervical dislocation, and gastric tissues were examined under a Leica stereomicroscope. In the normal control group, the gastric tissue appeared normal in color, with no ulcers or bleeding points. In contrast, the model group exhibited extensive bleeding on the stomach's inner wall, forming a continuous bleeding surface with severe erosion and ulceration. Compared with the model group, the ulcer severity in each treatment group was significantly reduced, with effects comparable to the positive drug group, demonstrating better therapeutic outcomes in ulcer healing.

Finally, gastric tissues were fixed in 10 % formaldehyde, embedded in paraffin, stained, and examined microscopically to obtain pathological images of gastric tissues from each group. As shown in Fig. 4e, the normal control group displayed evenly stained villi, intact cytoplasm, and uniformly sized and distributed cavities. In contrast, the model group showed severe cytoplasmic vacuolation in the villi, occasional necrosis such as nuclear pyknosis and rupture, and mild inflammatory cell infiltration. The size and distribution of villous cavities showed no significant change compared to the control. In the positive drug group, cytoplasmic vacuolation was significantly reduced, necrosis was absent, inflammatory cell infiltration was decreased, and the cavity size remained stable. Each treatment group showed a significant reduction in villous damage compared to the model group, with therapeutic effects equivalent to the positive drug.

Fig. 4f shows that in the normal control group, the muscle layer staining was uniform, cells were closely arranged, cytoplasm was intact, and muscle fibers were regular and clear. The model group exhibited uneven staining, marked vacuolation, and disorganized striations. The positive drug group displayed uniform staining and marked improvement in cytoplasmic vacuolation. The degree of muscle injury in the treatment groups was significantly lower than in the model group, with therapeutic effects similar to those of the positive drug (Fang et al., 2018; Wang et al., 2017; Yao et al., 2018).

## 4 Conclusion

In summary, Harigabri, a traditional Mongolian remedy for gastrointestinal ailments, exhibits a previously unrecognized nanostructure closely resembling GO. Using complementary analytical techniques—including Raman spectroscopy, XRD, Fourier-transform infrared spectroscopy (FT-IR), and TEM—both Harigabri and its ultrasonic extract were shown to possess the characteristic layered architecture and oxygen-containing functional groups of GO. Given the well-documented antibacterial and anticancer activities of GO, yet its limited clinical translation due to size- and surface-related biosafety concerns, our findings provide a crucial scientific rationale for advancing its therapeutic applications.

Indeed, Harigabri, its extract, and GO each demonstrated potent antibacterial activity against key gastrointestinal pathogens and significant efficacy in a gastric ulcer model. This work provides a solid scientific foundation for the clinical application of GO, effectively addressing major challenges in its therapeutic use. Furthermore, this study lays the groundwork for future innovations, potentially revolutionizing the treatment of gastrointestinal disorders and gastric cancer by integrating natural substances like Harigabri with graphene-based technologies.

## CRediT authorship contribution statement

**Galama Ang:** Writing – original draft, Formal analysis, Data curation, Conceptualization. **Qiaoli Ren:** Validation, Supervision, Methodology, Investigation. **Jun Ai:** Writing – original draft, Project administration, Funding acquisition. **Gerile Aodeng:** Resources, Methodology, Investigation.

## Declaration of competing interest

The authors declare that they have no known competing financial interests or personal relationships that could have appeared to influence the work reported in this paper.

## Data Availability

No data was used for the research described in the article.

## Appendix A Supplementary data

Supplementary material Image 1
