# Supplementary material for: Nanostructural insights into Mongolian medicine Harigabri and its therapeutic efficacy for gastrointestinal diseases
Source: Food Chem X. 2025 Jul 24;29:102838. doi: 10.1016/j.fochx.2025.102838 (PMC12318344; doi:10.1016/j.fochx.2025.102838)
Supplement: Supplementary file 1 — Supplementary material [file mmc1.pdf]

Certificate of Elsevier Language Editing Services

The following article was edited by Elsevier Language Editing Services:

Nanostructural Insights into Mongolian Medicine Harigabri and Its Efficacy in Treating Gastrointestinal Diseases

Ordered by:

Jun Ai

Estimated Delivery date:

2025-07-03

Order reference:

ASLESTD1107746
